# Using the Effect of Compression Stress in Fatigue Analysis of the Roller Bearing for Bimodal Stress Histories

**DOI:** 10.3390/ma15010196

**Published:** 2021-12-28

**Authors:** Paweł J. Romanowicz, Dariusz Smolarski, Marek S. Kozień

**Affiliations:** 1 Department of Machine Design and Composite Structures, Faculty of Mechanical Engineering, Cracow University of Technology, ul. Warszawska 24, 31-155 Kraków, Poland; pawel.romanowicz@pk.edu.pl; 2 Institute of Technology, State University of Applied Sciences in Nowy Sącz, ul. Staszica 1, 33-300 Nowy Sącz, Poland; dariuszsmolarski@interia.pl; 3 Department of Applied Mechanics and Biomechanics, Faculty of Mechanical Engineering, Cracow University of Technology, ul. Warszawska 24, 31-155 Kraków, Poland

**Keywords:** spectral method, multiaxial fatigue, mean stress effect, bimodal stresses, rolling contact fatigue, multiaxial high-cycle fatigue criteria, fatigue life

## Abstract

A new approach based on the direct spectral method for fatigue analysis of elements subjected to bimodal stress histories, including high compression effects, is proposed. A correction factor, taking into account the influence of the mean compressive stresses, is used in the proposed method. Equivalent amplitude is estimated, based on criteria proposed by Smith, Watson, and Tooper, and by Bergmann and Seeger. The method is presented with example of a thrust roller bearing. Two cases in which the rollers were subjected to constant force 206 N (where constant amplitude stresses occurred in the rollers) and cyclic force (where bimodal stresses with variable amplitudes occurred in the rollers) are studied. It is observed that multiaxial fatigue criteria (Crossland, Papadopoulos) do not include the influence of bimodal stresses and should not be used for such loading conditions. The proposed method includes both kinds of stress waveforms in the fatigue analysis and can be applied for the accurate identification of stress components and the determination of fatigue life. The damage rate calculated by the proposed approach for rollers subjected to a cyclic force (equivalent load equal to 151 N) was 0.86, which is in good agreement with the recommendations provided in the literature. The obtained accuracy of the proposed method is above 95%.

## 1. Introduction

Analyses of fatigue strength or remaining service life of mechanical components that are subjected to cyclic loading are essential for safety and economic reasons [1,2]. Fatigue failures are caused by random, repetitive, or cyclic loads that are generally significantly lower than loadings that would lead to the plastic deformation of the material. The onset of fatigue cracks may occur due to material imperfections, structural discontinuities, or stress concentrators [3,4,5,6,7,8]. The applied fatigue loads may be uniaxial (with one stress component), biaxial (with two stress components), or multiaxial (with more than two stress components) [9,10,11]. In the case of uniaxial random loadings, classical methods such as rainflow counting, cumulative damage models, and S-N curves can be used [10]. 

Fatigue analyses of structures subjected to complex multiaxial loadings can be made with the use of multiaxial high-cycle fatigue criteria. Such models allow for reducing the complex cyclic stress state to the equivalent uniaxial fatigue stress state. A comprehensive review of such criteria can be found in previous articles [12,13,14]. Such fatigue hypotheses are based on different approaches (i.e., integral approaches, energy formulations, stress or strain invariants, critical plane approaches, and empirical approaches) [15], and most of them are limited to certain applications (e.g., materials, loading conditions, etc.) [13,15]. Despite the availability of many multiaxial high-cycle criteria, it is difficult to find a single universal model [16]. Another limitation of the existing criteria is that, while they can be applied for the analysis of mechanical parts in which cyclic constant amplitude stresses occur, in practical applications, random or quasi-random loads occur [9]. The application of the multiaxial high-cycle fatigue criteria for such loading conditions requires additional methods, such as the rainflow counting algorithm, to determine uniform alternating cycles for fatigue analysis [17]. 

An example of the problem in which a multiaxial stress state occurs is rolling contact fatigue. This phenomenon applies to crane wheels [15], railway wheels and rails [18,19], rolling bearings [20], gears [21], etc. With respect to the crane wheel example, it was shown that not all criteria are suitable for the analysis of elements working in rolling contact conditions [15]. The best agreement with experimental tests was obtained using criteria based on an integral approach (i.e., the Papadopoulos model) [22]. However, that model is suitable for constant amplitude stress distributions. In cases in which a mechanical part is subjected to more complex stresses, such as bimodal stresses with variable amplitudes [23], fatigue analysis requires different computational approaches. 

In the relevant literature, there are works in which dedicated models are built for fatigue analysis of structures that are subjected to bimodal vibrations. The first approach was proposed by Sakai and Okamura [24]. Their idea was to extend the pattern used for waveforms with a narrow frequency band associated with one frequency [25,26] to waveforms with two dominant frequencies, clearly spaced from each other. The disadvantage of their proposed approach was the lack of consideration of the increase in amplitude for the lower frequency waveform by the share of the second harmonic component of the process. 

Fu and Cebon [23] found that Sakai and Okamura’s [24] attempt did not account for amplitude magnification for the lower frequency process [26]. Therefore, Fu and Cebon proposed their own concept of the number of stress cycles, taking into account the superposition of the amplitudes of harmonic components. 

Benasciutti and Tovo [27] compared the above two methods and established a modified Fu-Cebon method. An interesting approach, provided by Jiao and Moan [28], was dedicated to processes in which there are two characteristic harmonic waveforms: one originating from forced vibrations having a fixed character, and the other originating from impulse-forced vibrations with a transient nature of damped vibrations. Han et al. [29] focused on the above-mentioned three methods: Jiao-Moan, Fu-Cebon, and modified Fu-Cebon. It has been observed that these three bimodal methods do not have analytical solutions. An analytical solution for predicting vibration fatigue-life in bimodal spectra was developed based on random attempts [29]. 

All of the mentioned approaches [23,24,25,26,27,28,29] for fatigue analysis of bimodal stress histories belong to the group of so-called spectral methods, in which frequency response is described by functions characterized by random waveforms, such as power spectral density and k-th power spectral density moments. The proposed approaches were generally based on the assumption of a superposition of two narrowband focused realizations with two dominant frequencies. The modeling differed fundamentally in the ways of describing the narrowbanded nature of the realizations, often by adopting an appropriate distribution of a random variable [23,24,25,26,27,28,29]. 

The approach used by the authors and discussed in this article is called the direct spectral method, due to the frequency analysis carried out in the time domain by describing a bimodal realization in the form of a superposition of two harmonic waveforms with specific frequencies and amplitudes. The description is therefore deterministic in formulation, although it is associated with two frequencies. The difference in the approaches of the spectral methods and the direct spectral method (as previously formulated and modified) may be analogous to the interpretation of the application of the Fourier transform and expansion into the Fourier series in the study of the variability of functions. An example of the application of the spectral methods to broadband analysis (including the attempts by Rayleigh, Dirlik, and Benasciutti-Tovo) was provided by Kozien and Nieslony [30].

The authors formulated the original method for cycle counting of bimodal stress histories of a completely reversed type, based on their spectral characteristics in the case of superposition of the completely reversed stress cycles (the direct spectral method). This original method was formulated for the simple case of uniaxial stress [31,32], and for multiaxial stress [33,34]. 

The next step in fatigue analysis is using the information of identified cycles, including their mean and amplitude values for the estimation of fatigue lifetime, which is realized by the application of the suitable rule of cumulative damage. The most popular rule in engineering applications is the Palmgren-Miner linear cumulative damage rule [35,36,37]. The advantages and limitations of its application have been known for many years. Therefore, different approaches taking into account the effects of cumulative nonlinear damage are considered, e.g., the double-linear damage rule, as well as models proposed by Marco and Starkey, Subramanyan, Hashin and Rotem, Corten and Dolon, Freudenthal-Heller, Serensen, Bui-Quoc [38], and Liou [26].

The proposed approach based on the modified direct spectral method was presented with the example of a cylindrical thrust roller bearing. The novelty of the proposed approach is that it makes possible the determination of fatigue strength and fatigue life for structures subjected to non-constant-amplitude stresses. This is made possible by extending the direct spectral method by the correction factor, including the influence of compressive stresses on admissible shear stress amplitude. In the performed analysis, the roller bearing was subjected to a cyclic force. This resulted in the appearance of complex stresses with variable amplitudes over time in a roller. The results obtained by the proposed method were verified with multiaxial high-cycle criteria. It was observed that the influence of bimodal stresses is not taken into account in the multiaxial high-cycle fatigue criteria, and the application of those criteria for such loading conditions may lead to an overestimation of fatigue stress. On the other hand, the proposed approach, based on the direct spectral method, yields more reliable results for such loading conditions.

The paper is divided into 4 sections. The current state-of-the-art and the introduction are set out in Section 1. The description of the material and the investigated roller bearing are described in Section 2.1. The mathematical formulation for calculating subsurface stresses in the case of rolling contact, as well as loading conditions, are presented in Section 2.2. The multiaxial high-cycle fatigue criteria (proposed by Papadopoulos and Crossland) are described in Section 2.3. The direct spectral method is described in Section 2.4. In Section 3, the results are presented and discussed. Loading conditions and identification of stress cycles for the analyzed case are presented in Section 3.1. The results obtained by the application of the multiaxial high-cycle criteria are described in Section 3.2; the problem of the influence of negative normal stresses on fatigue strength is also discussed. A proposed modification of the calculation of the equivalent stress amplitude is given in Section 3.3. The application of the proposed modified spectral method for the investigated cases is described in Section 3.4. Finally, the paper is concluded in Section 4.

## 2. Materials and Methods

### 2.1. Material 

**Figure 1:**
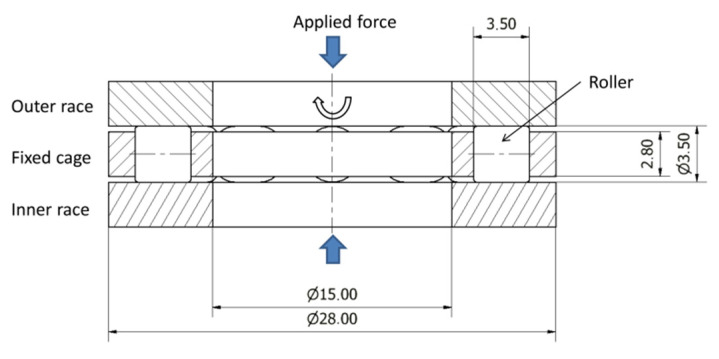
Geometry of K81102TN cylindrical roller thrust bearing and loading conditions applied in the analysis.

The presented analysis was carried out for a cylindrical roller thrust bearing designated as K81102TN [39]. The basic dynamic load rating of the selected bearing was 11.2 kN, and the fatigue load limit was 2.45 kN. The outside and bore diameters of the bearing were equal to 28 mm and 15 mm, respectively, and the roller diameter and length were 3.5 mm (Figure 1). Cylindrical rollers and rings of the bearing were made of AISI 52100 bearing steel. The chemical composition and the material properties of the material are presented in Table 1 and Table 2. 

**Figure 2:**
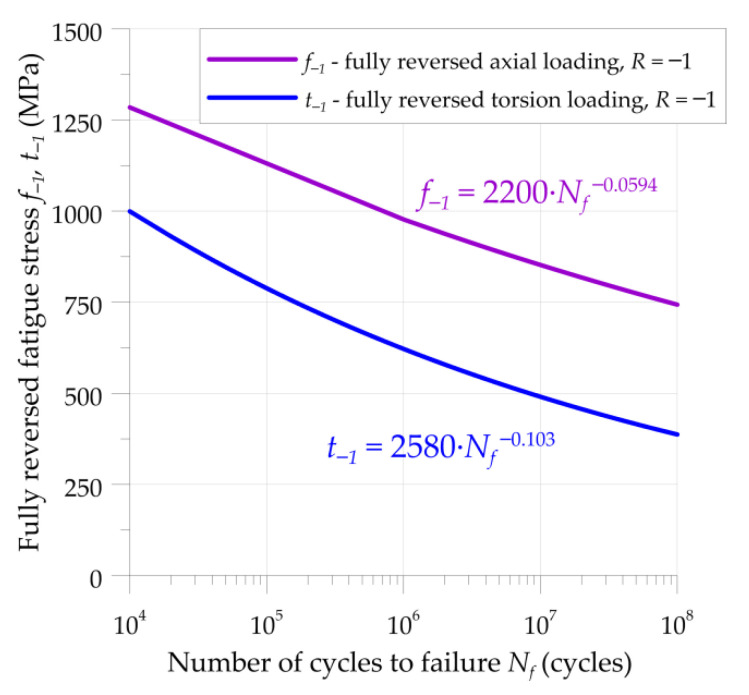
S-N curves for fully reversed axial *f*_−1_ and fully reversed torsion *t*_−1_ fatigue loadings, AISI 52100 bearing steel.

The fatigue analysis was made with the use of S-N curves. Generally, an analysis under multiaxial loading conditions requires the application of two typical S-N curves for fully reversed axial *f*_−1_ and fully reversed torsion *t*_−1_ fatigue loadings. The experimental fatigue tests for both of the above loading conditions were carried out and published by Shimizu et al. [41,42] and Saki [43]. On the basis of these studies, and with the use of Weibull distribution functions [41,44], the *f*_−1_ and *t*_−1_ S-N curves were determined for 10% failure life of the material, as indicated in Figure 2. It should be also noted that the high cycle fatigue tests [41,42,43] do not reveal fatigue limits for AISI52100 bearing steel. 

### 2.2. Loading Conditions

**Figure 3:**
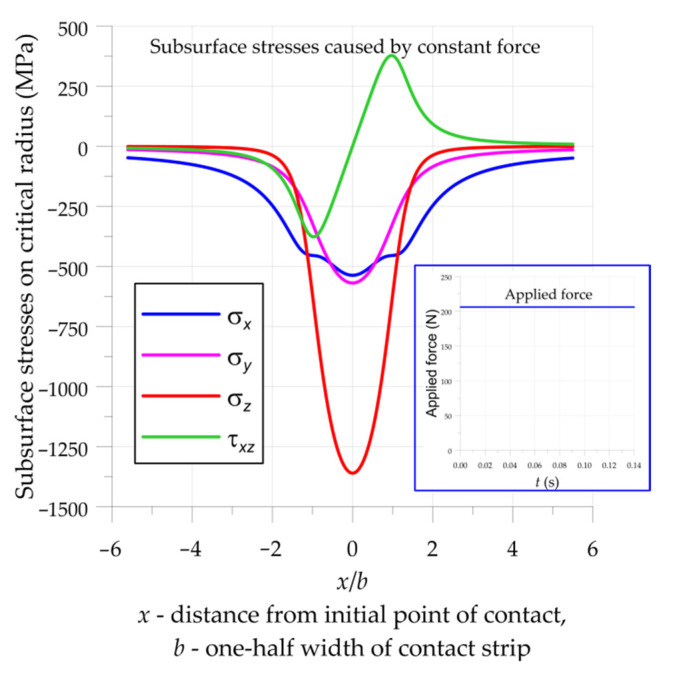
Subsurface stresses in the investigated cylindrical roller thrust bearing on critical radii in a bearing subjected to constant force (the force acting on one roller is *F* = 206 N).

The elements of rolling bearings were subjected to the cyclic multiaxial stresses caused by repeated contact between rollers and rings. The highest fatigue stresses were induced at a certain depth below the surface. In the investigated case (i.e., the cylindrical roller thrust bearing), the subsurface stresses could be calculated by using the solution of the line contact of two cylinders pressed together [45]. For this purpose, the elliptic coordinates α-β related to the *x*-*z* coordinate system, with the following dependencies:

(1) $$\{\begin{array}{c} x=b\cdot \cosh\left(\alpha \right)\cdot \cos\left(\beta \right)\;\;\\ z=b\cdot \sinh\left(\alpha \right)\cdot \sin\left(\beta \right) \end{array},$$

where *x* is the horizontal direction of rolling, *y* is the direction along the contact line, and *z* is the vertical direction (in which the compression of rollers occurs). Finally, the stresses on the planes perpendicular to the coordinate axes could be calculated as follows:

(2) $$\left\{ \begin{array}{c} \sigma_{x}=-\frac{2q}{\pi \cdot b}e^{-\alpha }\sin\left(\beta \right)+\frac{2q}{\pi \cdot b}\sin\left(\beta \right)\cdot \sinh\left(\alpha \right)\cdot \left[1-\frac{\sinh\left(2\alpha \right)}{\cosh\left(2\alpha \right)-\cos\left(2\beta \right)}\right]\\ \sigma_{y}=-\frac{2q}{\pi \cdot b}\cdot \frac{\lambda }{\lambda +\mu }e^{-\alpha }\sin\left(\beta \right)\\ \sigma_{z}=-\frac{2q}{\pi \cdot b}e^{-\alpha }\sin\left(\beta \right)-\frac{2q}{\pi \cdot b}\sin\left(\beta \right)\cdot \sinh\left(\alpha \right)\cdot \left[1-\frac{\sinh\left(2\alpha \right)}{\cosh\left(2\alpha \right)-\cos\left(2\beta \right)}\right]\\ \tau_{xz}=-\frac{2q}{\pi \cdot b}\sinh\left(\alpha \right)\cdot \sin\left(\beta \right)\cdot \frac{\sin\left(2\beta \right)}{\cosh\left(2\alpha \right)-\cos\left(2\beta \right)}\\ \tau_{xy}=\tau_{yz}=0 \end{array}\right.$$

where *q* is the loading per unit length of the contact area and λ and μ are Lame’s constants. Typical distributions of the subsurface stresses caused by a constant force acting on a bearing are presented in Figure 3. The stresses are indicated in relation to a dimensionless parameter *x*/*b*, related to the contact size (*x* is a distance to the initial point of contact, and *b* is a one-half the width of the contact area). Verification of the mathematical formulations was made by the finite element method presented in earlier studies [46,47]. Assuming that the elements of bearing (i.e., the rollers and rings) are in relative motion, and considering the rotational speed of the bearing, the presented distributions in Figure 3 may be treated as distributions of stresses, in time, for one loading cycle.

Such distributions can be characterized by the high 3-dimensional compression stresses and the characteristic in-phase shift between normal and shear stresses. Both effects have a significant influence on fatigue life [48,49,50]. Such loading conditions require the application of fatigue criteria, which consider the distribution of the whole stress tensor in time. 

### 2.3. Multiaxial High-Cycle Fatigue Criteria

The analysis of rolling contact fatigue can be made using multiaxial high-cycle fatigue criteria [11,12,13,15,22,49,51]. Based on the practical applications, two criteria (i.e., the Crossland and the Papadopoulos criteria) were selected for the present study [15,20,46,47,48,49,50]. The Crossland model [51] includes the linear combination of the amplitude of the second stress invariant σ_vM,a_ and the maximal value of the first stress invariant σ_H,max_ (additional details are provided in Appendix A), as set out in the formula below:

(3) $$\tau_{C}=\frac{\sigma_{\mathrm{vM},a}}{\sqrt{3}}+a_{C}\cdot \sigma_{H,\max}\leq t_{-1}$$

where

(4) $$a_{C}=\{\begin{array}{c} 0\;\;\;\;\;\;\mathrm{for}\;\;\;\frac{3t_{-1}}{f_{-1}}\leq \sqrt{3}\;\;\\ \frac{3t_{-1}}{f_{-1}}-\sqrt{3}\;\;\;\;\;\;\;\mathrm{for}\;\;\;\frac{3t_{-1}}{f_{-1}}>\sqrt{3} \end{array}$$

and *f*_−1_ and *t*_−1_ are the fully reversed bending and torsion fatigue limits, respectively.

The criterion proposed by Papadopoulos [22] was based on the integral approach, and took into account the amplitude of the resolved shear stress τ_Δ,a_ (see Appendix A) and the maximal value of the first stress invariant σ_H,max_, as shown by the formula below:

(5) $$\tau_{P1}=\sqrt{\frac{5}{8\pi^{2}}\overset{2\pi }{\underset{\varphi =0}{\int }}\overset{\pi }{\underset{\theta =0}{\int }}\overset{2\pi }{\underset{\chi =0}{\int }}\tau_{∆,a}^{2}(\varphi ,\theta ,\chi )d\chi \cdot \sin(\theta )d\theta d\varphi }+a_{C}\cdot \sigma_{H,\max}\leq t_{-1}$$

The Papadopoulos hypothesis is proposed for hard metals and provides good compatibility with experimental rolling contact fatigue tests [20,48]. Additional details about the application of both criteria were considered in previous studies [15,20].

### 2.4. The Modified Direct Spectral Method

The direct spectral method is generally focused on cases of cycle counting of bimodal processes when stress variation is due to vibrations of the structure. Generally, this occurs when an oscillating process is the reason for the kinematic or force type of excitation.

The direct spectral method is based on the following assumptions:

- the low-frequency component varies with frequency ω_1_;
- the low-frequency component has a generally higher amplitude (half the difference between the maximal and minimal values), which is constant (*A*_1_ = const);
- the high-frequency components vary with frequency ω_2_; and
- the high-frequency components may need to vary in amplitude time *A*_2_(*t*).

A limitation in using the direct spectral method, in comparison to the other spectral methods used to analyze the bimodal realizations, is related to the general ideas behind these methods. The direct spectral method is deterministic, and the other spectral methods are random. Thus, if the process is bimodal and the data are a form of a time series (i.e., they are data in the time domain), it will be natural to use the direct spectral method. On the other hand, if the available data are defined in the frequency domain, e.g., as a power spectral density function, the natural approach will be to use one of the other spectral methods. In such a case, the direct method requires an additional process of identifying the amplitudes for the two deterministic frequencies, which was carried out by the authors in a previous study [32].

Originally, the direct spectral method was formulated for completely reversed stress histories of harmonic types, in both primary and secondary cycles [31,32,33,34,35]. For the analyzed case of rolling bearing fatigue, the method’s algorithm was slightly modified due to the fact that the primary cycle was not generally harmonic (i.e., the shape did not have the sine type), and three of the four non-zero stress components had a pulsating type. As stress variation in time is well-known for bearings in dangerous regions near contact, a decision was made to identify the cycles directly on the basis of the available stress component histories in the time domain. 

The algorithm for application of the method can be stated in the following steps:

- identify the frequencies ω_1_ and ω_2_ of the bimodal stress history, based on the stress histories in the time domain;
- obtain the values corresponding to the frequencies for periods *T*_1_ and *T*_2_, where period *T*_1_ is the base period for the stress signal;
- define the stress block period *T_B_* based on the values of *T*_1_ and *T*_2_, where *T_B_* is the smallest total multiple of the *T*_1_ period for which the ratio *T_B_*/*T*_1_ is the approximate integer;
- the primary stress cycle has a stress amplitude equal to the stress mean value, as follows:

(6) $$\sigma_{a}=\sigma_{m}=\left[A_{1}+\underset{0\leq t\leq T_{B}}{\max}A_{2}\left(t\right)\right]$$

- the amplitudes of the secondary stress cycles have varying-in-time amplitudes, σ_a_(*t*) = *A*_2_(*t*), associated with the stress mean values that are the values of the function varying with frequency ω_1_ for moments of time that are the middle values of subsequent periods of secondary function, with frequency ω_2_ for 0 ≤ *t* ≤ *T_B_*;
- based on the obtained values for the identified cycles (i.e., mean and amplitude values), the values of the equivalent stress are calculated for the basic cycle and the secondary cycles using the Von Mises hypothesis;
- the obtained data describing the identified stress cycles for a given waveform are the basis for further fatigue analysis, using the chosen stress cumulation hypothesis, i.e., Palmgreen-Miner’s [36,37,38,52,53,54].

## 3. Results and Discussion

### 3.1. Loading Conditions and Identification of Stress Cycles for the Analyzed Case

**Figure 4:**
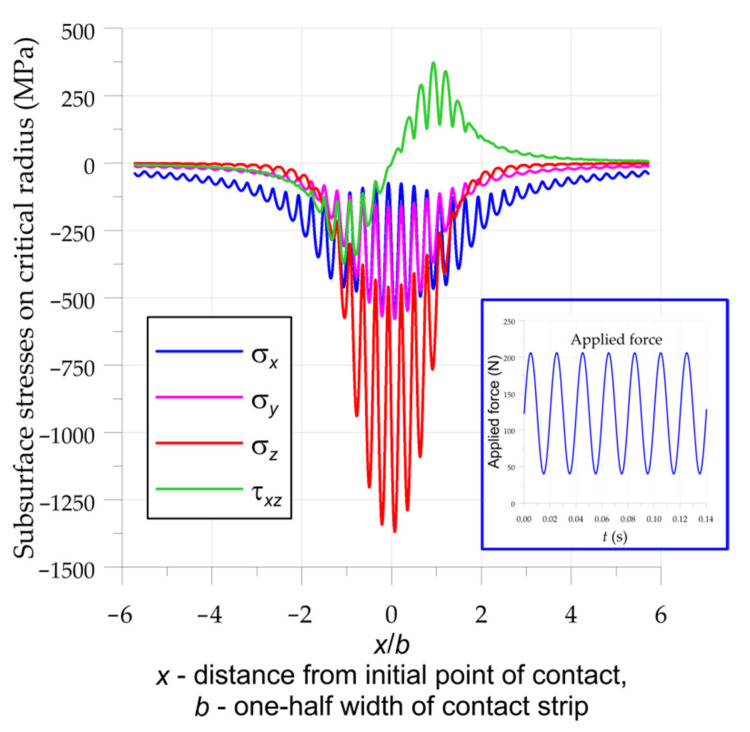
Subsurface stresses in the investigated cylindrical roller thrust bearing on critical radii in the bearing subjected to cyclic force (force acting on one roller: *F*_m_ = 123 N, *F*_a_ = 83 N).

The investigated cylindrical thrust roller bearing was subjected to the cyclic pulsating force (Figure 1). The maximal force acting on bearing was equal to the fatigue load limit 2.45 kN given in the manufacturer’s catalogue [39]. More information about the determination of the fatigue load in the bearing is available from a previous study [20]. The assumed force acting on one roller had an amplitude of *F*_a_ = 83 N and a constant mean value, *F*_m_ = 123 N (Figure 4).

The calculations were performed for the frequency of the force, set in such a way that for a period *x* = 2*b* (the time of rolling through the contact area) seven full cycles of force were realized (Figure 4). Subsurface stresses (Figure 4) were calculated with the use of the formulations given by Equation (2). Fatigue loading was determined based on the assumption that the fatigue life of the bearing should be equal to 1 million rotations of the bearing at a 90% rating life. It corresponded to *N_f_* = 1.2×10^7^ cycles in rollers and rings. The fully reversed bending fatigue limit *f*_−1_ and the fully reversed torsion fatigue limit *t*_−1_ could be determined from the Wöhler’s curves given in Figure 2: they were equal to *t*_−1_ = 480 MPa and *f*_−1_ = 835 MPa.

The identified non-zero stress components in the roller (i.e., the stress mean values σ*_x_*_,m_, σ*_y_*_,m_, σ*_z_*_,m_, τ*_xz_*_,m_) and the stress amplitudes (σ*_x_*_,a_, σ*_y_*_,a_, σ*_z_*_,a_, τ*_xz_*_,a_) for primary and secondary cycles are shown in Table 3. The identification was done by application of the described direct spectral method, as discussed in Section 2.4. For the analyzed case, *T_B_* = *T*_1_ and *T*_1_ = 40∙*T*_2_. *T*_1_ is the time of contact of the roller. It should be noted, however, that due to the realistic distribution of stress in the roller, the base cycles for normal stress (σ*_x_*, σ*_y_*, σ*_z_*) have the form of pulsating compression with period *T*_1_, and the cycle for tangential stress (τ*_xz_*) has the form of a fully reversed type with period *T*_1_.

The obtained values of stress amplitudes and mean values for identified primary cycle and the secondary ones were used for further fatigue life analysis, as described and discussed in Section 3.4.

It should be noted that by applying the modified direct spectral method to the simulated time histories of the actual changes of the components of the stress tensor, it became possible to accurately identify all secondary cycles (with higher frequency and lower amplitudes), and the base cycle (with a lower frequency and maximum amplitude) resulting from the cycle with lower frequency ω_1_, lower amplitudes, and higher frequency ω_2_ (a conservative approach). Although another part of the analysis of the damage rate of the element showed that the secondary cycles have a very small impact on fatigue life, that factor does not change the generality of the method’s possibilities for various cases of bimodal time variability. If one of the spectral methods were used for fatigue analysis, it would be necessary to determine the corresponding power spectral density function on the basis of the stress realizations in the time domain, and then carry out fatigue analysis. Due to the variability of the secondary cycle amplitudes, in the case of determining the power spectral density function for the given realizations, amplitude of this function may be reduced due to the leakage effect for the analyzed frequency ω_2_.

### 3.2. Multiaxial High-Cycle Fatigue Criteria

**Figure 5:**
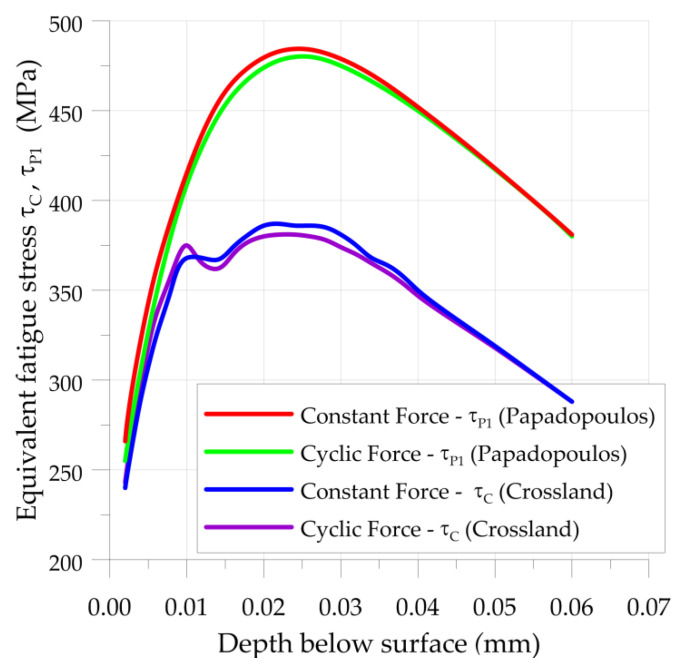
Fatigue stresses on different depths, calculated by Crossland and Papadopoulos criteria; K81102TN roller bearing subjected to constant and cyclic force.

The calculations of the fatigue strength of the K81102TN roller bearing were made for two cases with constant force (Figure 3) and pulsating force (Figure 4). First, the critical depths in which the highest fatigue stress occurs were determined (Figure 5). 

Subsurface stresses were determined with the use of the analytical solutions described in Section 2.2 (Equations (1) and (2)). Fatigue stresses were estimated by two different multiaxial high-cycle fatigue criteria (Crossland (3) and the Papadopoulos (5)). Due to the analysis of subsurface rolling contact fatigue, the calculations were made from a depth of 0.002 mm. The maximal fatigue stress was determined at a depth 0.026 mm below the surface of the roller (Figure 5). The influence of surface roughness was not included in the analysis. The subsurface stresses in machine parts subjected to the rolling contact can be described by the following distinctive features (Figure 3):

- three-dimensional pulsating high compression stresses;
- fully reversed shear stresses; and
- in-phase shift between normal compressive and shear stresses.

**Figure 6:**
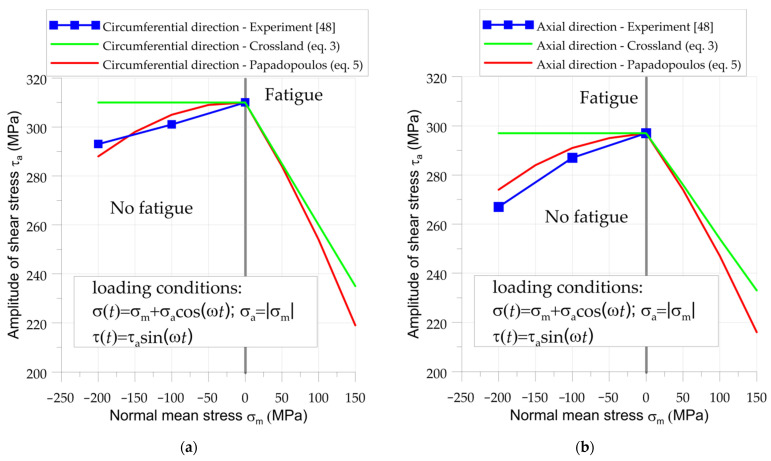
Comparison of results obtained from Crossland and Papadopoulos criteria, with experimental tests for samples extracted from railway wheel made of R7T steel: (**a**) samples cut in circumferential direction; (**b**) samples cut in axial direction. Experimental results are taken from Ref [48].

All of the above phenomena have a significant influence on the fatigue strength of the element subject to rolling contact fatigue. Both multiaxial criteria (Crossland and Papadopoulos) consist of two parts. The first is related to the equivalent cyclic shear stress; the second is related to the maximal value of the hydrostatic stress, σ_H,max_. In the investigated case the maximal value of hydrostatic stress, σ_H,max_, is equal to 0. The experimental tests of non-proportional out-of-phase fully reversed torsion (τ_a_ > 0, τ_m_ = 0) and pulsating compression (σ_a_ > 0, σ_m_ = −σ_a_) carried by Bernasconi et al. [48] revealed that an increase in compressive stresses results in a reduction of the allowable shear stress amplitude. Such tests were carried out with samples extracted from a railway wheel made of R7T steel [55]. Due to slight anisotropy, the results obtained by Bernasconi et al. [48] were presented for samples extracted from the wheel in two different directions, circumferential (Figure 6a) and axial (Figure 6b). The determined fatigue limits for both cases (5–7 samples were tested for each loading condition [48]) and a comparison of the calculation results using multiaxial high-cycle fatigue hypotheses are presented in Figure 6. This comparison is very important because most of the multiaxial high-cycle fatigue criteria assume the favorable influence of compressive stresses on fatigue [15]. It was observed that criteria based on the integral approaches (such as Papadopoulos’s model) are most compatible with the experimental results for rolling contact fatigue. Other criteria may strongly underestimate fatigue stress, due to overestimation of the influence of high compressive stresses [15,48]. This effect can be seen in the example of the Crossland hypothesis (Figure 6), which does not take into account the influence of compressive stress on equivalent fatigue stress. 

Summarizing the above results, it can be seen that the Crossland hypothesis overestimates fatigue strength and should not be used for rolling contact fatigue analysis. The Papadopoulos criterion provides a good estimation of fatigue strength when a bearing is subjected to constant force. However, Papadopoulos’s criterion is not able to include cyclic force in the fatigue analysis, for two reasons: integral formulation is used in the Papadopoulos model and, because of this, the Papadopoulos criterion is proposed for constant amplitude stresses. Therefore, application of the Papadopoulos model requires additional time-consuming analysis with the use of, e.g., rainflow counting and cumulative damage models.

### 3.3. Eqivalent Stress Amplitude

The influence of mean stress in the proposed modified direct spectral method is included by the use of equivalent stress amplitude, σ_a,eqv_. The equivalent amplitude is estimated on the basis of the criteria proposed by Smith, Watson, and Tooper [56], and Bergmann and Seeger [57,58,59].

(7) $$\sigma_{a,\mathrm{eqv}}=\sqrt{\left(\sigma_{a}+k\cdot \sigma_{m}\right)\sigma_{a}}$$

where σ_m_ is the mean stress, σ_a_ is the stress amplitude, and *k* is a correction factor. The value of the correction factor depends on the sensitivity of the material to the mean stress. 

The application and verification of the above parameter in the fatigue analysis of mechanical components subjected to cyclic loading with mean normal stresses was demonstrated in previous studies [60,61,62,63].

### 3.4. The Modified Direct Spectral Method 

The modified direct spectral method is an extension of the direct spectral method, proposed by the authors for the analysis of bimodal fatigue cases. This method appears to be a natural way of identifying and counting cycles, especially when the time histories of stress variation in time are known. The presented modification includes the influence of compression on fatigue life. The method is an alternate approach for fatigue analysis in bimodal cases, for which the statistical/random attempt seems to be less natural. In the analyzed case, the identification of stress amplitude and mean values was carried out in an analytical way. Moreover, the value of *T_B_* was easy to determine, due to the fact that *T*_1_/*T*_2_ = 40. When the known characteristics of stress are in the form of power spectral density, the proposed method can be applied [32] by a much more natural way than a random attempt.

It was observed that the Papadopoulos multiaxial high-cycle fatigue hypothesis gives the most accurate estimation of the fatigue strength of structures working under rolling contact conditions and subjected to constant force [15,20,46,47,48,49,50]. Because of this, the calibration of the proposed model was made with the use of the Papadopoulos criterion. Such calibration was made for roller bearing subjected to constant force. For such a purpose in the first step, the critical constant force acting on one roller, *F* = 206 N (Figure 3), was determined with the use of the Papadopoulos model. It should be noted that this value is compatible with the fatigue limit stated in the manufacturer’s catalogue [39]. In the second step, the calculations were made via the application of the proposed modified spectral method, and the correction factor was fitted in such a way that equivalent fatigue stress was equal to the fatigue limit. The determined value of the correction factor was equal to *k* = 0.68.

Fatigue analysis was performed for both cases described in the paper. In the first case, one roller of the thrust bearing K81102TN was subjected to constant force *F* = 206 N. In this case, only one cycle was observed for each particular stress (No 1.1 in Table 4).

In the second case (No. 1.2 in Table 4), applied force on one roller of the bearing was cyclic, with mean value *F*_m_ = 123 N and amplitude *F*_a_ = 83 N. Mean values and amplitudes of stress components in a roller subjected to cyclic force are set out in Table 3. The equivalent stress amplitudes were calculated by Formula (7), with *k* = 0.68 for all determined components. The damage rate, *D_MDSM_*, was determined by the linear cumulative damage (Palmgren-Miner’s) rule [54]. It was observed that the highest impact on the damage rate was the primary cycle. Accordingly, only the primary cycle is reported in Table 4. However, the damage rate *D_MDSM_* was calculated on the basis of including all the components reported in Table 3 (i.e., the primary cycle 1 and the secondary cycles, 1–40).

The value of the damage rate in the case of the modified direct spectral method was calculated with the following formula:

(8) $$D_{MDSM}=\underset{i}{\sum }\frac{n_{i}}{N_{i}}=\frac{n_{i}}{{\left(\frac{\sigma_{\mathrm{vMF},i}}{2200}\right)}^{\frac{1}{-0.0594}}},$$

where *n_i_* is the number of applied cycles, *N_i_* is the number of cycles to failure, and σ_vMF,i_ is the equivalent fatigue stress for the *i*-th component calculated by the Von Mises formula.

With respect to multiaxial high-cycle fatigue criteria, the damage parameter was estimated as follows:

(9) $$D_{MHCF}=\frac{\tau_{C}}{t_{-1}}\;\;\;\mathrm{or}\;\;D_{MHCF}=\frac{\tau_{P1}}{t_{-1}},$$

where *t*_−1_ is the fully reversed torsion fatigue strength for the assumed number of cycles to failure.

The value of the damage rate in the case of roller bearing subjected to the constant force is obviously equal to *D_MDSM_* = 1 (No. 1.1 in Table 4, the fatigue limit determined from the S-N curve is equal to *f*_−1_ = 835 MPa), because this scenario was used for calibration of the proposed modified spectral method.

In the second case, the maximal value of applied force (*F_max_* = *F*_m_ + *F*_a_ = 123 N + 83 N = 206 N) was equal to the constant force applied in the first case. When the multiaxial high-cycle criteria were applied, the fatigue damage of roller subjected for constant and cyclic force was almost the same (see, e.g., compare the results No. 2.1 and 2.3 for Crossland, and compare the results No. 2.2 and 2.4 for Papadopoulos in Table 5, Figure 5). This fact is incompatible with the information provided in the manufacturer’s catalog [39]. Moreover, the Crossland hypothesis underestimates the damage rate by neglecting the influence of compressive stresses.

In contrast to both multiaxial high-cycle fatigue criteria, the proposed modified spectral method considered the reduction of the damage rate in rollers caused by the application of cyclic force (see, e.g., the differences in results No. 1.1 and 1.2 in Table 4 when compared with results No. 2.2 and 2.4 in Table 5). The estimated damage rate under cyclic force with the use of the modified spectral method was equal to *D_MDSM_* = 0.86. This trend, obtained by the proposed approach, is in good agreement with the recommendations proposed by the manufacturer [39]. Using the formula proposed in [39], the equivalent mean load acting on the roller subjected to cyclic force, with the magnitude of the load constantly varying between a minimum value *F*_min_ and a maximum value *F*_max_, is equal to:

(10) $$F_{\mathrm{eqv}}=\frac{F_{\min}+2F_{\max}}{3}≅151N$$

Further verification of the obtained damage rate for cyclic force (see No. 1.2 in Table 4) was made by comparing this result with the damage parameter calculated according to the Papadopoulos criterion for the constant equivalent mean force, *F*_eqv_ = 151 N (No. 2.5 in Table 5). Clearly, high accuracy of the proposed modified direct spectral method (above 95%) was obtained.

In summary, application of the multiaxial high-cycle fatigue criteria is not recommended for the direct analysis of bearings subjected to a cyclic force. If multiaxial high-cycle fatigue criteria are applied, additional analyses, such as rainflow counting, must also be used. More reliable results are obtained by applying the modified spectral method.

## 4. Conclusions

The modified spectral method was proposed in this paper and successfully applied to the analysis of a roller bearing subjected to constant and cyclic force. Based on this study, the following conclusions can be drawn:

1. Multiaxial high-cycle fatigue criteria cannot be applied for an analysis in which stresses do not have constant amplitude waveforms. Moreover, only a few multiaxial high-cycle criteria may be applied to problems in which high negative stresses occur.
2. The proposed approach based on the modified spectral method considers the mean value of stresses (as well as negative normal stresses). It makes possible the determination of fatigue strength and fatigue life for structures working in different loading conditions, including machine parts working in the regime of high negative normal stresses (i.e., rolling contact fatigue).
3. The obtained results by the modified spectral method are in good agreement with the multiaxial high-cycle criteria, when constant-amplitude stresses are applied.
4. Due to the possibility of identifying mean values and amplitudes of stress components, the proposed method makes possible the determination of fatigue life when a mechanical part is subjected to complex or bimodal stress histories.
5. Basing on the manufacturer’s recommendation, the results obtained by the application of the modified spectral method to a roller bearing are more reliable than results obtained by multiaxial high-cycle fatigue criteria. The high accuracy of the proposed method (above 95%) in comparison with the Papadopoulos criterion was noted.
6. For unknown stress histories in the time domain (i.e., lack of knowledge regarding the bimodal spectrum) or for irregular histories, the spectral analysis must be applied. For non-stationary stress histories, especially in cases with a variable in time amplitudes, the Short-time Fourier transform (STFT) may be applied to identify stress amplitudes in time. In such cases, the first part of fatigue cycle identification (i.e., finding amplitudes and mean values of stress components) can be carried out as presented in previous articles [31,32,33,34]. Further analysis, especially in regard to estimating equivalent stresses, may be led by the proposed approach.

## Figures and Tables

**Table 1:** Chemical composition of bearing steel AISI 52100 (in wt%) [40].

C	Cr	Mn	Si	P	S
0.95–1.05	1.30–1.65	0.25–0.45	0.15–0.35	<0.027	<0.025

**Table 2:** Mechanical properties of bearing steel AISI 52100 [40].

Yield Limit	Tensile Strength	Hardness	Fracture Toughness
2000 MPa	2250 MPa	60–67 HRC	15.4–18.7 MPa·m^1/2^

**Table 3:** Identified mean values and amplitudes of stress components.

Cycle Type	No.	σ*_x_*_,m_ MPa	σ*_y_*_,m_ MPa	σ*_z_*_,m_ MPa	τ*_xz_*_,m_ MPa	σ*_x_*_,a_ MPa	σ*_y_*_,a_ MPa	σ*_z_*_,a_ MPa	τ*_xz_*,_a_ MPa
Primary cycle	1	−281.4	−289.9	−684.7	0	281.4	289.9	684.7	373
Secondary cycles	1	−39.9	−12.5	−1.7	−7.5	8.4	2.4	0.4	0.6
	2	−43	−13.5	−1.9	−8.4	8.6	2.4	0.5	0.8
	3	−46.5	−14.7	−2.3	−9.5	8.8	2.4	0.7	1
	4	−50.7	−16	−2.7	−11	9.2	2.5	0.9	1.4
	5	−55.7	−17.7	−3.4	−12.7	9.6	2.5	1.3	1.7
	6	−61.6	−19.7	−4.2	−15	10.3	2.6	1.7	2.3
	7	−68.7	−22.2	−5.3	−17.8	11.2	2.7	2.3	2.9
	8	−77.5	−25.3	−6.9	−21.5	12.6	2.8	3.2	3.8
	9	−88.2	−29.5	−9.3	−26.5	14.7	3.3	4.5	4.8
	10	−101.6	−35	−12.9	−33.3	18	4.2	6.3	6.2
	11	−118.8	−42.4	−18.6	−43	23.8	5.6	9.1	7.8
	12	−140.4	−52.5	−27.8	−57.7	32.8	7.9	13.4	10.2
	13	−166.7	−66.7	−43.4	−82.5	47	12.1	19.3	15.4
	14	−200.6	−87	−72.3	−122.1	71.5	19.5	27.5	24.3
	15	−240.6	−116.4	−144.4	−170.9	108.9	32.5	50.6	44.2
	16	−276.5	−155.9	−287.7	−213.3	151.7	53	92.7	72.3
	17	−285.8	−215.8	−460.1	−255.3	176.1	92.8	160	117.7
	18	−284.9	−282.8	−658.6	−227	192	141.7	281.1	114.7
	19	−298.8	−335.3	−819	−150.5	218.5	181	385.2	82.1
	20	−314.4	−364.6	−901.3	−56.1	239.8	204.4	441.4	55
	21	−319.7	−369	−910.3	52.5	243.2	210.7	459.2	53.5
	22	−312.2	−348.5	−849.5	148.5	226.5	199.9	439.9	56.7
	23	−298.2	−304.2	−716.1	208.6	197.3	171.5	374.9	81.5
	24	−292.3	−241	−513.1	257.2	174.5	127.8	253.7	115.3
	25	−291	−173.6	−295.1	238	161.5	80.3	113.8	102,5
	26	−261.9	−121.4	−147.4	173.5	130.4	45.9	50.4	57.3
	27	−217.3	−87.6	−73.6	120.6	92.2	26.8	23.3	27.2
	28	−177.5	−66.2	−39.7	83.3	63.6	16.8	11.2	15.4
	29	−146.4	−51.9	−25.6	58.3	45	11.5	8.2	9.6
	30	−122.6	−42.1	−17.3	42.4	33.3	8.4	5.8	6.2
	31	−104.3	−34.9	−12.1	32.1	25.8	6.5	4.2	4.1
	32	−90	−29.6	−8.7	25.2	20.9	5.4	3.1	3
	33	−78.7	−25.6	−6.5	20.5	17.5	4.6	2.3	2,4
	34	−69.6	−22.4	−5	17	15.2	4.1	1.7	1.9
	35	−62.2	−19.9	−4	14.3	13.6	3.7	1.2	1.5
	36	−56.1	−17.8	−3.2	12.2	12.3	3.4	0.9	1.2
	37	−51.1	−16.1	−2.6	10.5	11.5	3.2	0.7	0.9
	38	−46.8	−14.7	−2.2	9.2	10.8	3.1	0.6	0.7
	39	−43.2	−13.5	−1.8	8.3	10.2	3	0.4	0.7
	40	−40.1	−12.5	−1.6	7.5	9.8	2.9	0.3	0.7

**Table 4:** Results obtained by using the modified spectral method (primary cycles) for a roller bearing subjected to constant and cyclic force.

No.	Force	σ*_x_*_,a,eqv_ MPa	σ*_y_*_,a,eqv_ MPa	σ*_z_*_,a,eqv_ MPa	τ*_xz_*_,a,eqv_ MPa	σ_vMF_ MPa	*D_MDSM_* ^1^
1.1	Constant *F* = 206 [N]	346.1	367.8	879.4	376.2	835	1
1.2	Cyclic *F*_m_ = 123 [N], *F*_a_ = 83 [N]	364.7	375.7	887.5	373.0	827.7	0.86

^1^ The fatigue limit is equal to the fully reversed bending fatigue strength *f*_−1_ = 835 MPa.

**Table 5:** Results obtained by using the multiaxial high-cycle fatigue criteria for a roller bearing subjected to constant and cyclic force.

No.	Force	Criterion	τ_C_ MPa	τ_P1_ MPa	*D_MHCF_ =* τ_C_/*t*_−1_^1^	*D_MHCF_ =* τ_P1_/*t*_−1_^1^
2.1	Constant *F* = 206 [N]	C	387	-	0.81	-
2.2	Constant *F* = 206 [N]	P1	-	480	-	1
2.3	Cyclic *F*_m_ = 123 [N], *F*_a_ = 83 [N]	C	382	-	0.80	-
2.4	Cyclic *F*_m_ = 123 [N], *F*_a_ = 83 [N]	P1	-	475	-	0.99
2.5	Constant *F* = 151 [N]	P1	-	415	-	0.87

^1^ The fatigue limit is equal to the fully reversed torsion fatigue strength *t*_−1_ = 480 MPa.

## Data Availability

The data presented in this study are available on request from the corresponding author.

## Nomenclature

*A*_1_	amplitude of the low-frequency component
*A*_2_	amplitude of the high-frequency component
*a_C_*	constant of Crossland and Papadopoulos criteria
*B*	one-half width of the contact area
*D*	damage rate parameter
*f*_−1_	fully reversed axial/bending fatigue strength
*F*	constant force applied on one roller
*F*_a_	amplitude of force applied on one roller
*F*_eqv_	constant equivalent mean force acting on one roller
*F*_m_	mean value of force applied on one roller
*k*	correction factor
*n_i_*	number of applied cycles of i-th component
*N_i_*	number of cycles to failure for i-th component
*N_f_*	number of cycles to failure
*R*	stress ratio
*q*	loading per unit length of contact area
*t*	time
*t*_−1_	fully reversed torsion fatigue strength
*T*_1_	base period for the stress signal
*T*_2_	secondary period for the stress signal
*T_B_*	stress block period
*x*	horizontal direction of rolling,
*y*	direction along the contact line,
*z*	vertical direction
α-β	elliptic coordinates
λ and μ	Lame’s constants
σ_a,eqv_	equivalent amplitude of stress
σ_1_, σ_2_, σ_3_	principal stresses
σ_i_	(*i = x,y,z,xz*) stress component
σ_i,m_	mean value of stress component
σ_i,a_	amplitude of stress component
σ_vM,a_	amplitude of the second stress invariant
σ_vMF,i_	equivalent fatigue stress for i-th component calculated by the Von Mises formula.
σ_H,max_	maximal value of hydrostatic stress
τ_a_	amplitude of the shear stress
τ_Δ,a_	amplitude of resolved shear stress
Δ	material plane defined by angles ψ and θ
Χ	direction of shear stress on plane Δ
τ_C_	fatigue stress calculated by Crossland hypothesis
τ_P1_	fatigue stress calculated by Papadopoulos hypothesis
ω_1_	frequency of low-frequency component
ω_2_	frequency of high-frequency component

## Appendix A

The amplitude of the second stress invariant is calculated as follows:

(A1) $$\sigma_{\mathrm{vM},a}=\underset{t}{\max}\left[\frac{1}{\sqrt{2}}\sqrt{{\left(\sigma_{x,a}-\sigma_{y,a}\right)}^{2}+{\left(\sigma_{y,a}-\sigma_{z,a}\right)}^{2}+{\left(\sigma_{z,a}-\sigma_{x,a}\right)}^{2}+6\left(\tau_{xy,a}{}^{2}+\tau_{xy,a}{}^{2}+\tau_{xy,a}{}^{2}\right)}\right]$$

where σ_i,a_ is the amplitude function of stresses and is varying in time [15] and calculated as follows:

(A2) $$\sigma_{i,a}=\sigma_{i,a}\left(t\right)=\sigma_{i}\left(t\right)-\sigma_{i,m}$$

and σ*_i_*(*t*) is the distribution of stress in time, and σ*_i,_*_m_ is the mean value of stress.

The maximal value of hydrostatic stress is calculated as:

(A3) $$\sigma_{H,\max}=\underset{t}{\max}\left(\frac{\sigma_{1}\left(t\right)+\sigma_{2}\left(t\right)+\sigma_{3}\left(t\right)}{3}\right).$$

The amplitude of resolved shear stress τ_Δ,a_ (ψ,θ,χ) for specified material plane Δ defined by angles ψ and θ (χ is the direction of shear stress on plane Δ) is calculated as follows:

(A4) $$\tau_{∆,a}\left(\varphi ,\theta ,\chi \right)=0.5\left[\underset{t}{\max}\tau \left(\varphi ,\theta ,\chi ,t\right)-\underset{t}{\min}\tau \left(\varphi ,\theta ,\chi ,t\right)\right].$$
